# Cited2 inhibited hypoxia-induced proliferation and migration of PASMCs via the TGF-β1/Cited2/PPARγ pathway

**DOI:** 10.22038/IJBMS.2023.74455.16178

**Published:** 2024

**Authors:** Hong-Juan Wang, Lan Ma, Qin Yu

**Affiliations:** 1 The First School of Clinical Medicine, Lanzhou University, Lanzhou 730000, Gansu, China; 2 Department of Respiratory Medicine, Gansu Provincial Hospital, Lanzhou 730000, Gansu, China; 3 Department of Plateau Medical Center, Qinghai University, Xining 810000, Qinghai, China; 4 Department of Respiratory Medicine, The First Hospital of Lanzhou University, Lanzhou 730000, Gansu, China

**Keywords:** Cited2, Hypoxia-induced - pulmonary hypertension, Migration, Proliferation, Pulmonary artery smooth - muscle cells

## Abstract

**Objective(s):**

Proliferation and migration of pulmonary artery smooth muscle cells (PASMCs) contribute to hypoxia-induced pulmonary hypertension (HPH). The transcription factor Cbp/p300-interacting transactivator with Glu/Asp-rich carboxy-terminal domain 2 (Cited2) has been implicated in the control of tumor cells and mesenchymal stem cell (MSC) and cardiomyocyte growth or migration. Whether Cited2 is involved in the proliferation and migration of PASMCs and the underlying mechanisms deserve to be explored.

**Materials and Methods:**

Cited2 expression was detected in rat PASMCs under hypoxia conditions and HPH rat models. The effect of Cited2 on the proliferation and migration of PASMC was detected by overexpression or knockdown of the Cited2 gene. After PAMSCs were treated with recombinant TGF-β1 and the lentivirus vector overexpressing Cited2, expression of peroxisome proliferator-activated receptor gamma (PPARγ) was examined by western blotting.

**Results:**

We revealed that hypoxia down-regulated the expression of Cited2 in PASMCs and rat pulmonary arteries. Cited2 overexpression inhibited the proliferation and migration of PASMCs under hypoxia, while Cited2 knockdown induced the proliferation and migration of PASMCs. Cited2 inhibits the negative regulation of the TGF-β1 pathway on PPARγ to inhibit the proliferation and migration of PASMCs.

**Conclusion:**

These findings suggest that increased Cited2 expression contributes to the inhibition of PASMCs proliferation and migration by regulating TGF-β1-mediated target gene expression in HPH and provides a new target for molecular therapy of HPH.

## Introduction

Pulmonary hypertension (PH) is a progressively worsening disorder, which is characterized by continued elevation of pulmonary artery pressure, accompanied by progressive pulmonary vessel remodeling, which results in subsequent right heart failure (1). Hypoxia-induced pulmonary hypertension (HPH) is related to lung diseases and/or hypoxia and regulated by a multitude of molecular pathways and processes (2). It has been demonstrated that pulmonary vasoconstriction, vascular remodeling, and erythrocytosis caused by hypoxia were important contributors to HPH (3). Abnormal proliferation and migration of pulmonary artery smooth muscle cells (PASMCs) are present within the remodeled pulmonary vessels (4, 5). Although PH treatment is challenging, the guidelines do not recommend existing effective drugs to treat HPH. Hence, the development of novel approaches to improve the worsening outcome and shortened survival in HPH is required.

CBP/p300-interacting transactivator with glutamic acid/aspartic acid-rich carboxylterminal domain 2 (Cited2), which belongs to the Cited family, is critical for organ development, cell growth and differentiation, and metabolic homeostasis and immunity (6-8). At the cellular level, Cited2 was found to have a pro-proliferative effect in cardiac stem cells and HEK293 cells (8, 9). However, Cited2 overexpression in cardiomyocytes, cardiac endothelial cells and neuronal cells suppresses cell proliferation (10-12), revealing that Cited2 has a unique function in cell proliferation. At the molecular level, Cited2 lacks a DNA binding domain but exhibits a high affinity for the CH1 domain of the transcriptional co-activator CBP/p300 (13). By binding to the CH1 domain of CBP/ p300, the Cited2 protein regulates the CBP/p300-mediated transactivation of multiple transcription factors, such as HIF-1α, P53, TFAP2, STAT, PPARα, and PPARγ (14-18). So, researchers found that Cited2 may play important roles in cancers, cardiovascular disease, and other metabolic diseases.

In recent years, much research has been devoted to inhibiting the activity of HIF to treat PH (19, 20), but with limited success. Cited2 is a negative regulator of HIF1 by competing to bind to the same binding domain (CH1) of CBP/p300, and it has a 33-fold stronger and tighter binding capacity compared to HIF-1α (21). Cited2 is a very efficient switch of the hypoxia response, and it is likely to be crucial in HPH.

As is well known, transforming growth factor-beta (TGF-β) is involved in pulmonary vascular remodeling and PH development, and PASMCs are important targets of TGF-β (22, 23). Cited2 could be down-regulated by TGF-β in different cells (24, 25) through post-transcriptional regulation (26). However, Cited2 could also modulate TGF-β-mediated up-regulation of VEGFA and MMP9 in human breast cancer cell lines (27, 28). These studies revealed that Cited2 may modulate the TGF-β1 signaling network. However, whether TGF-β1 also down-regulates Cited2 and thus is implicated in the PASMC proliferation and migration by this pathway in hypoxia remains unclear. If this assumption could be clarified, it may be a meaningful mode of action in HPH therapy. So, the effects of Cited2 in PASMC proliferation and migration were investigated in this study, and the underlying molecular mechanisms were further explored.

## Materials and Methods

***Animals***

The Animal Care Committee of Gansu Provincial Hospital approved all animal experimentation protocols (Approval number: 2023-595). Sprague-Dawley (SD) rats (male, 180-220 g) were purchased from Beijing Vital River Laboratory Animal Technology Co., Ltd. Twelve rats were randomized into two groups: hypoxia group (n=6) and normoxia group (n=6). A hypobaric hypoxia chamber with 10% oxygen was used to maintain the hypoxia group for 4 weeks. The normoxia group was kept in room air (21% oxygen) for the same time as normal controls. The 12-hour cycles of light and darkness, as well as food and water, were made freely available to all rats. Four weeks later, the mean pulmonary arterial pressure (mPAP), right ventricular hypertrophy index [(right ventricle (RV)/(left ventricle (LV)+septum weight (S), RVHI], as well as pulmonary artery wall thickness were measured to confirm successful modeling.

***Hematoxylin and eosin (HE) staining***

Rat lung tissues were preserved for 48 hr at room temperature in 4% paraformaldehyde to create paraffin sections, and then the paraffin blocks were cut into sections of 5 μm thickness. The pulmonary arterioles’ wall thickness was measured with HE staining on the sections. After HE staining was routinely performed, the following formula was used to determine the percentage of the pulmonary arteriole’s mean wall thickness (WT%):

WT% = 100 × wall thickness/vessel semi-diameter.

The following formula was used to determine the percentage of the total area that was made up of the pulmonary artery wall (WA%):

WA%=100×cross-sectional area of the wall area/total cross-sectional area of the vessel.

***Immunohistochemical (IHC) staining***

For the detection of Cited2 protein in rat lungs, the manufacturer’s instructions of the immunohistochemistry kit purchased from Solarbio were followed for performing IHC staining on the paraffin sections. The primary antibody used was anti-Cited2 (1:100, Affinity). After being dewaxed to water, antigen retrieval was performed on the slices using an antigen retrieval solution. Then, the endogenous peroxidase was eliminated using a 3% H_2_O_2_ solution. After being blocked with goat serum, the paraffin sections were reacted with primary antibody overnight at 4 ^°^C. On the second day, the sections were reacted with biotin-labeled secondary antibody at 37 ^°^C for 1 hr. After developing these sections using a DAB solution, the nuclei were then stained with hematoxylin. 

***Immunofluorescence (IF)***

The primary antibodies used in IF to study the expression and localization of Cited2 or identify the PASMCs were anti-Cited2 (1:50, Santa Cruz) and anti-a-smooth muscle actin antibody (anti-α-SMA, 1:300, Abcam). After being fixed for 40 min by using 4% paraformaldehyde, the PASMCs were incubated for 15 min by using 0.3% Triton X-100. Afterward, goat serum was used to block the cells for 1 hr, followed by incubation with Cited2 antibody overnight at 4 ^°^C. On the following day, CoraLite594-labeled goat anti-mouse secondary antibody (1:100, Proteintech) was used to incubate the cells for 1 hr at room temperature in the dark. Hoechst 33342 (1:1000, Solarbio) was used to stain the nuclei for 3 min, and then a fluorescence microscope was used to observe the cells.

***PASMC isolation***

The explant method as previously reported (29) was used successfully in the present study to separate primary PASMCs from healthy adult SD rats (180-220 g). The pulmonary arteries were placed in Dulbecco’s modified Eagle’s medium (DMEM, Gibco) with 20% fetal bovine serum (FBS, Cell-box) at 37 ^°^C and 5% CO_2_. IF was performed to identify the PASMCs with anti-α-SMA (1:300, Abcam). After cell passaging, DMEM with 12% FBS was used to culture the PASMCs. The subsequent experiments utilized passages of 2 to 5 cells.

***Hypoxia exposure of PASMCs***

Serum-free DMEM was used for cell starvation for 24 hr before all experiments. A hypoxia incubator (1% O_2_, 94% N2, and 5% CO_2_) was used to culture the cells of the hypoxia group for 0, 24, 48, and 72 hr, separately. Meanwhile, an incubator with 21% O_2_, 74% N2, and 5% CO_2_ was used to culture the cells of the normoxia group. 

***Cell infection***

The lentivirus vector overexpressing Cited2 and the corresponding no-load lentiviral vector were constructed by Hanbio. The lentiviral vector containing siRNA fragments for inhibiting Cited2 and the corresponding no-load lentiviral vector were constructed by Genechem. According to the directions provided by the manufacturer, lentiviral particles were transfected into PASMCs. They were appended to the medium for 24 hr. For an additional 24 hr, the media with lentivirus were switched out for DMEM containing 12% FBS. Then, the PASMCs were incubated in hypoxia for the subsequent experiments.

***5-Ethynyl-20-deoxyuridine (EDU) assay***

PASMC proliferation was detected using the EDU incorporation assay kit purchased from Beyotime, and EDU was performed in accordance with the manufacturer’s instructions.

***Transwell migration assay***

PASMCs were infused into the Transwell chamber’s upper chamber (Corning) after being resuspended in serum-free DMEM. Media containing 20% FBS filled the lower chamber. After being cultured for 24 hr in a hypoxia incubator, cells were fixed for 30 min with 4% paraformaldehyde. Cotton swabs were used to wipe off the non-migrating cells on the upper layer of the chamber membrane. Then, 0.1% crystal violet was used to stain the cells that migrated through for 20 min. Five views per well were selected at random at 100x magnification to count the number of stained cells.

***Quantitative reverse transcription-polymerase chain reaction (qRT-PCR)***

Total RNA was isolated from pulmonary arteries or cells by using TRIzol reagent (Invitrogen) in accordance with the manufacturer’s instructions. cDNA was synthesized using a reverse transcription kit (Tiangen). qPCR was performed using the SYBR Green Realtime PCR Master Mix (Tiangen). β-actin was used as an internal reference. The specific oligonucleotide primers were as follows: Cited2: forward, 5′-CCGCCCAATGTCATAGACACTGATTTC-3′ and reverse, 5′-ATTTCTTTCAGCCGCGAGGTTAACC-3′; β-actin: forward, 5′-CCTAAGGCCAACCGTGAAAA-3′ and reverse, 5′-CAGAGGCATACAGGGACAACAC-3′.

***Western blot***

Tissues and cells were extracted in RIPA lysis buffer (R0010, Solarbio) containing PMSF and a phosphatase inhibitor cocktail (P1045, Beyotime) to gain protein. The protein concentration was detected by using the bicinchoninic acid (BCA) protein assay kit (Solarbio). Total tissue or cellular lysate was transferred onto a 0.22 m polyvinylidene difluoride membrane (Solarbio) after being separated by 12% SDS-PAGE. A protein-free quick blocking solution (Boster, AR0041) was used to block the membrane at room temperature for 1 hr, and then primary antibodies against Cited2 (1:500, Affinity), anti-PPARγ (1:2000, Proteintech), and anti-β-actin (1:3000, Affinity) were used 

to incubate the membranes overnight at 4 ^°^C. On the next day, HRP-labeled secondary antibody (1:5000, Affinity) was used to incubate the membranes at room temperature for 1 hr, and the chemiluminescence method was used for color development.

***Statistical analysis***

The statistical analysis was performed with GraphPad Prism 8.0. Data were expressed as the mean±SEM. Statistical analysis was performed by independent-sample t-test for two groups. *P*<0.05 was considered statistically significant.

## Results

***Successful establishment of HPH rat model and primary culture of rat PASMCs***

**Figure 1:**
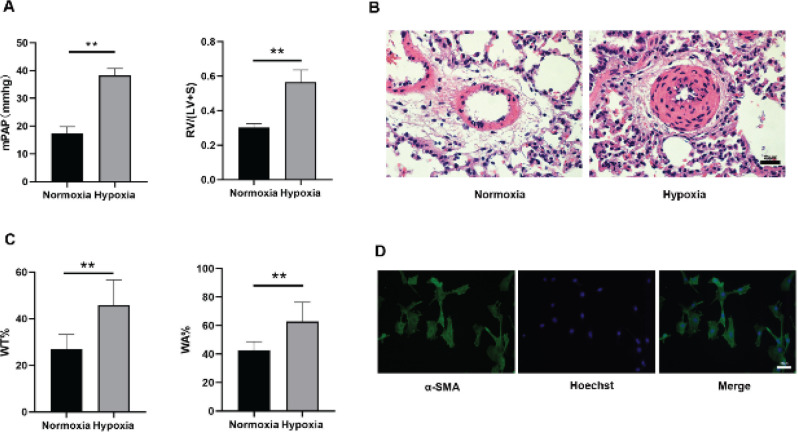
Hypoxia exposure induced pulmonary hypertension and pulmonary arterial remodeling

Male SD rats were randomized into hypoxia (n=6) and normoxia groups (n=6). For 4 weeks, the hypoxia group was exposed to 10% O_2_, whereas the normoxia group was housed at 21% O_2_. After being fed for 4 weeks under hypoxic or normoxic conditions, the RV/ (LV+S), mPAP, and wall thickness of the pulmonary arterioles were measured to assess whether the HPH rat model was successfully established. These results displayed that the mPAP and RV/ (LV+S) of HPH rats increased by comparison with the normoxia group (Figure 1A). Similarly, the wall thickness of the pulmonary arterioles and the wall area were much greater by comparison with the normoxia group, whereas the lumen diameter was significantly reduced (Figure 1B, C). The PASMCs isolated from the pulmonary arterioles of healthy adult rats were observed using a fluorescence microscope, and the results showed that the green fluorescence after actin was used bound to anti-α-SMA (Figure 1D). These results indicated that the HPH rat models were established with success, and the primary rat PASMCs were successfully isolated.

***Cited2 was down-regulated in the HPH rat model***

**Figure 2:**
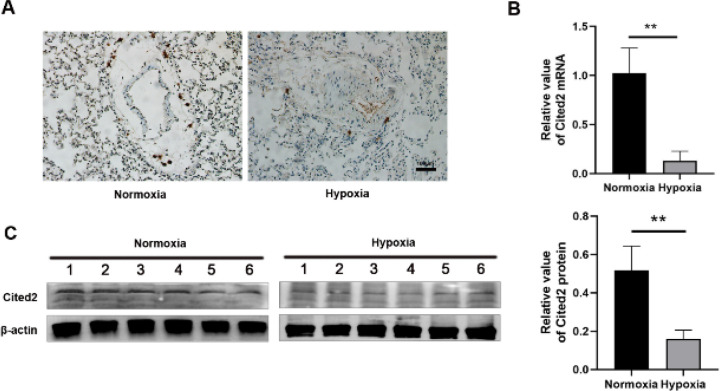
Cbp/p300-interacting transactivator with Glu/Asp-rich carboxy-terminal domain 2 (Cited2) expression was down-regulated in hypoxia-induced pulmonary hypertension (HPH) rat models

In most adult tissues and macrophages, hypoxia could down-regulate Cited2 expression (30, 31), which regulates cell proliferation and senescence via regulating the transactivation of various transcription factors mediated by CBP/p300. However, up to now, the biological role of Cited2 in HPH remains unknown. For clarification of this issue, the effect of hypoxia treatment (4 weeks) on Cited2 expression was examined in HPH rat pulmonary arteries. The analysis of Cited2 expression by IHC demonstrated that the hypoxia group had markedly decreased Cited2 protein levels (Figure 2A) by contrast with the normoxia group. The total RNA or protein was isolated from the pulmonary arteries of the hypoxia and normoxia groups. The results of Cited2 mRNA (Figure 2B) and protein level (Figure 2C) displayed similar results to the IHC experiment. The results revealed that Cited2 may participate in the development of HPH.

***Cited2 was down-regulated by TGF-β1 in hypoxic PASMCs***

**Figure 3:**
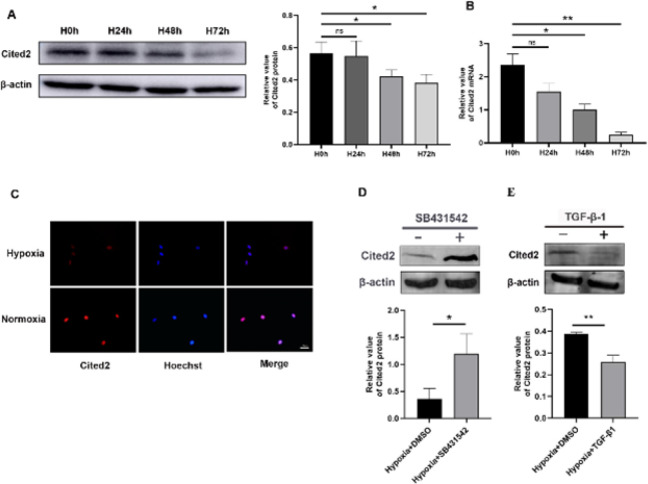
Cbp/p300-interacting transactivator with Glu/Asp-rich carboxy-terminal domain 2 (Cited2) expression was down-regulated in pulmonary artery smooth muscle cells (PASMCs) in hypoxia

PASMCs were presented in hypoxia for 0, 24, 48, and 72 hr (1% O_2_) separately to further determine the Cited2 expression in these cells in hypoxia. The outcomes displayed that at the protein and mRNA levels, hypoxia also inhibited Cited2 expression in PASMCs (Figures 3A and B). Cited2 expression was further confirmed by IF, which also displayed that Cited2 was located in the nucleus and down-regulated by hypoxia (Figure 3C). This observation revealed that Cited2 may have an important role in regulating cellular responses to hypoxia.

Cited2 was clarified to be down-regulated by hypoxia in the PASMCs and HPH rat model, but the mechanism was unclear. TGF-β1 could negatively regulate Cited2 in breast cancer cells, epithelial cells, and leiomyoma cells (26, 32, 33), and TGF-β1 expression is up-regulated in hypoxia, which is related to pulmonary vascular remodeling and the PH development (22, 34). In the present study, PASMCs were treated with SB431542 (TGF-β1 inhibitor, 10 gmol/l) for 24 hr in hypoxia and after serum starvation for 24 hr to further explore whether TGF-β1 is also involved in the regulation of Cited2 in PASMCs in hypoxia. The results demonstrated that SB431542 treatment increased the Cited2 protein expression in PASMCs in hypoxia (Figure 3D), indicating that inhibiting the TGF-β1 pathway could restore the expression of Cited2 in hypoxia. To further confirm that TGF-β1 modulates the expression of Cited2, TGF-β1 (10 ng/ml) was used in PASMCs in hypoxia. We also observed that 24 hr after TGF-β1 stimulation, the Cited2 protein level was down-regulated (Figure 3E). This result confirmed that Cited2 is a TGF-β1-responsive gene in hypoxia.

***Cited2 overexpression inhibited proliferation and migration of PASMCs in hypoxia***

**Figure 4:**
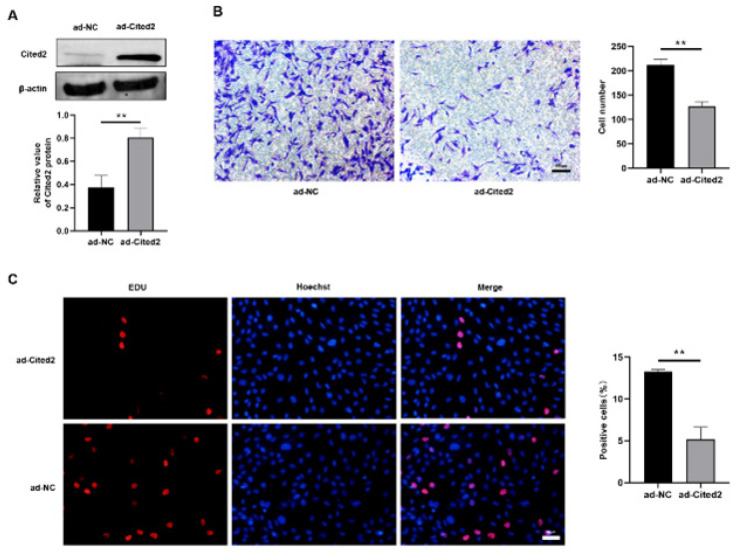
Effects of Cbp/p300-interacting transactivator with Glu/Asp-rich carboxy-terminal domain 2 (Cited2) overexpression on pulmonary artery smooth muscle cells (PASMCs)

For demonstration of the above assumption, the Cited2 overexpressed by lentivirus carrying Cited2 gene (*ad-Cited2)* and negative control (*ad-NC)* were transfected into PASMCs, and the levels of Cited2 were assessed at protein level (Figure 4A). The Transwell migration assay showed that compared with ad-NC, ad-Cited2 suppressed the PASMCs migration (Figure 4B) in hypoxia. According to the result of the EDU assay, the quantity of EDU-positive cells (Figure 4C) in the ad-Cited2 group decreased by contrast with the ad-NC group. These results revealed that Cited2 overexpression reversed hypoxia-induced cell proliferation and migration, which contributed to vascular remodeling in HPH. 

***Cited2 down-regulation induced proliferation and migration of PASMCs in hypoxia***

**Figure 5:**
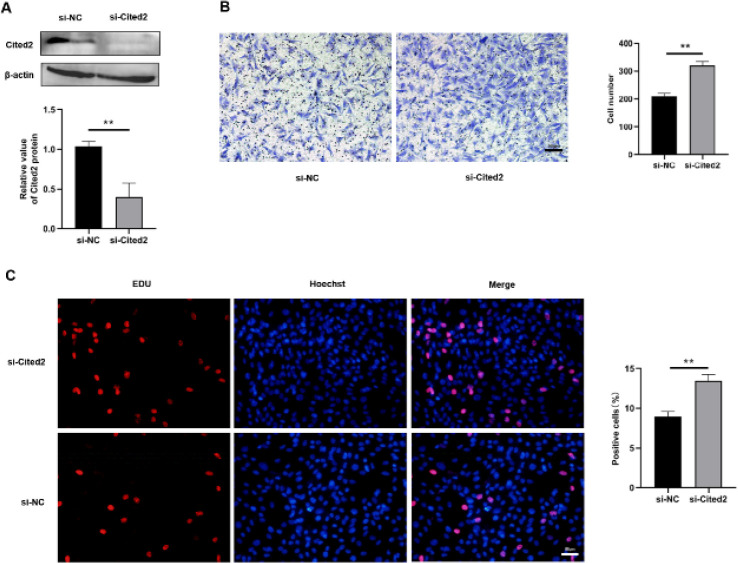
Effects of Cbp/p300-interacting transactivator with Glu/Asp-rich carboxy-terminal domain 2 (Cited2) knockdown on pulmonary artery smooth muscle cells (PASMCs)

The above experiments confirmed that overexpression of Cited2 could inhibit the proliferation and migration of PASMCs in hypoxia. For further understanding of the function of Cited2 in PASMCs proliferation and migration, Cited2 knockdown (*si-Cited2)* was performed in hypoxia, and Cited2 expression was evaluated at the protein level (Figure 5A). According to the result of EDU assay, the quantity of EDU-positive cells (Figure 5B) in the si-Cited2 group was elevated by contrast with the negative control (*si-NC)* group. The Transwell migration assay also displayed that si-Cited2 promoted the migration of PASMCs (Figure 5C) by contrast with si-NC in hypoxia. These results indicated that Cited2 knockdown induced the proliferation and migration of PASMCs in hypoxia. 

***Cited2 inhibited the negative regulation of the TGF-*** ***β*** ***1 pathway on PPAR*** ***γ*** ***to inhibit the proliferation and migration of PASMCs***

**Figure 6:**
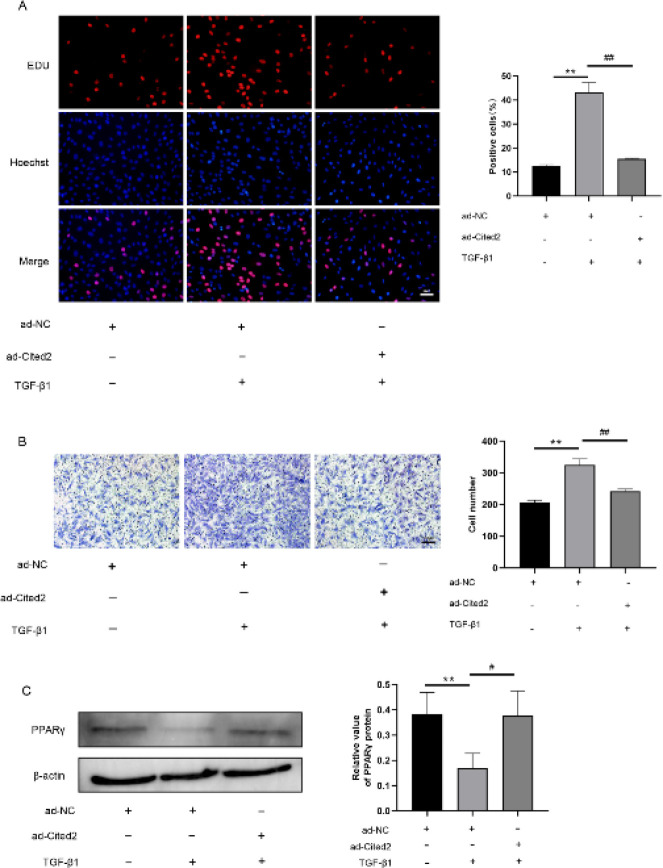
Cbp/p300-interacting transactivator with Glu/Asp-rich carboxy-terminal domain 2 (Cited2) reversed Transforming growth factor-beta1 (TGF-β1)-induced proliferation and migration of pulmonary artery smooth muscle cells (PASMCs)

This study demonstrated that Cited2 inhibited PASMCs proliferation and migration, and TGF-β1 played a key regulatory role in Cited2 expression in hypoxia. TGF-β1 (10 ng/ml) was used in Cited2-overexpressed PASMCs after serum starvation for 24 hr to further confirm that TGF-β1 may promote cell proliferation and migration by inhibiting Cited2. These results displayed that the pro-proliferation and pro-migration effects of TGF-β1 on PASMCs were reversed by (Figures 6A and B) Cited2 overexpression, suggesting that TGF-β1 may promote cell proliferation and migration by inhibiting Cited2.

Peroxisome proliferator-activated receptor-y (PPAR-γ) has been shown to prevent hypoxia-induced pulmonary vascular remodeling, indicating that PPAR-γ has a vasoprotective role under chronic hypoxic conditions (35). By inhibiting PPAR-γ expression at the transcriptional level, TGF-β1 mediates hypoxia-induced PPAR-γ down-regulation (36), whereas the TGF-β1 pathway was inhibited by PPARγ activation in human PASMCs (37). Cited2 has been proven to activate PPARγ (10, 38). In the present study, whether Cited2 inhibits the negative regulation of the TGF-β1 pathway on PPARγ to inhibit PASMC proliferation and migration was explored. Through the use of TGF-β1 recombinant protein (10 ng/ml) in Cited2-overexpressed PASMCs, TGF-β1 was found to inhibit the expression of PPARγ, but Cited2 overexpression resulted in the recovery of PPARγ expression (Figure 6C). This result indicated that Cited2 regulated PASMC proliferation and migration by mediating the negative regulation of the TGF-β1 pathway on PPARγ.

***PPARγ cooperates with Cited2 to inhibit the proliferation and migration of PASMCs***

**Figure 7:**
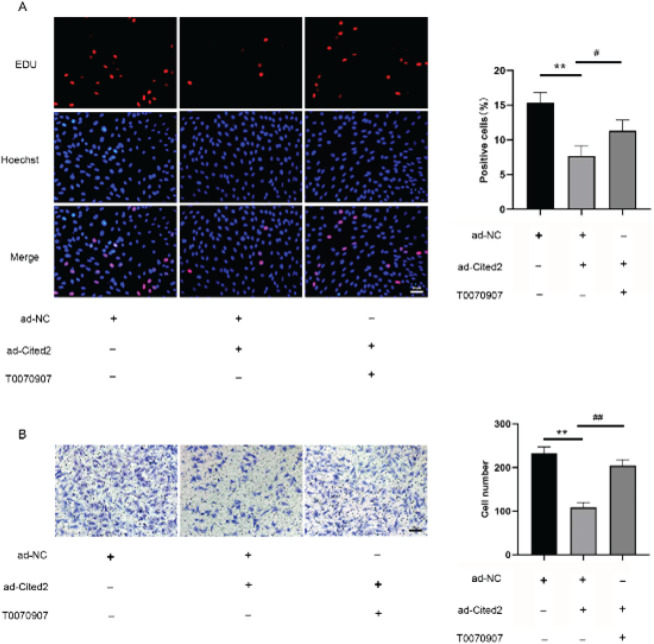
Peroxisome proliferatoractivated receptor gamma (PPARγ) cooperates with Cbp/p300-interacting transactivator with Glu/Asp-rich carboxy-terminal domain 2 (Cited2) to inhibit the proliferation and migration of pulmonary artery smooth muscle cells (PASMCs)

PPARγ are nuclear receptors with transcriptional activity that have been implicated in altered gene expression and cell signaling in PH pathogenesis (39). Interestingly, Cited2 is known to be a coactivator of PPARγ (10, 38). To better demonstrate that PPARγ cooperates with Cited2, PPARγ inhibitor T0070907 (10 μM) was used in Cited2-overexpressed PASMCs in hypoxia. The results demonstrated that the anti-proliferation and anti-migration effect of Cited2 on PASMCs were reversed by the inhibition of PPARγ (Figures 7A and B), suggesting that PPARγ cooperates with cited2 to inhibit cell proliferation and migration.

(A) Right ventricle (RV)/(left ventricle (LV)+septum weight (S) (RV/ (LV+S)) and the mean pulmonary arterial pressure (mPAP) of rats in normoxia (n=6) and hypoxia(n=6) groups.(B) Hematoxylin and eosin(HE) staining of lung tissues in normoxia (n=6) and hypoxia (n=6) groups. (C) Percentage of wall thickness of pulmonary arteries (WT) and wall area (WA) in normoxia (n=6) and hypoxia (n=6) groups. (D) Isolation and culture of primary PASMCs and identification of PASMCs by anti-α-SMA antibody. Data are means ± SEM. **P<0.01 vs normoxia group

(A) Protein expression in rat pulmonary arteries measured by immunohistochemistry (n=6). (B) mRNA expression in rat pulmonary arteries measured by quantitative reverse transcription-polymerase chain reaction (qRT-PCR)(n=6). (C) Protein expression in rat pulmonary arteries measured by Western blot (n=6). Scale bar, 100 μm. Data are means ± SEM. **P<0.01 vs normoxia group

(A) PASMCs were exposed to hypoxia for 0, 24, 48 and 48 hr, respectively, and Cited2 protein expression in PASMCs measured by Western blot (WB)(n=3 experiments). (B) PASMCs were exposed to hypoxia for 0, 24, 48 and 48 hr, respectively, and Cited2 mRNA expression in PASMCs measured by quantitative reverse transcription-polymerase chain reaction (qRT-PCR)(n=3 experiments). (C) Cited2 expression of PASMCs was examined by immunofluorescence after being exposed to normoxia or hypoxia for 48 hr (n=3 experiments). (D) Effects of SB431542 on protein expressions of Cited2 in PASMCs in hypoxia. PASMCs were exposed to hypoxia for 48 hr, and in the last 24 hr, SB431542 was used to treat PASMCs (n=3 experiments). (E)Transforming growth factor-beta1 (TGF-β1) modulates the expression of Cited2 in PASMCs in hypoxia. PASMCs were exposed to hypoxia for 48 hr, and in the last 24 hr, TGF-β1 was used to treat PASMCs (n=3 experiments). Data are means±SEM, *P<0.05, **P<0.01 vs contral

(A) Expression of Cited2 protein was detected in ad-NC and ad-Cited2 groups (n=3 experiments). (B) Transwell migration experiment to detect the migration of PASMCs in ad-NC and ad-Cited2 groups in hypoxia (n=3 experiments). (C) 5-Ethynyl-20-deoxyuridine assay (EDU) assays to detect the proliferation of PASMCs in ad-NC and ad-Cited2 groups in hypoxia (n=3 experiments). ad-Cited2: Cited2 overexpressin, ad-NC: negative control. Data are means±SEM, **P<0.01 vs ad-NC

(A) Expression of Cited2 protein was detected in si-NC and si-Cited2 groups (n=3 experiments). (B) Transwell migration experiment to detect the migration of PASMCs in si-NC and si-Cited2 groups in hypoxia (n=3 experiments). (C) 5-Ethynyl-20-deoxyuridine assay (EDU) assays to detect the proliferation of PASMCs in si-NC and si-Cited2 groups in hypoxia (n=3 experiments). si-Cited2: Cited2 knockdown. si-NC: negative control. Data are means±SEM, **P<0.01 vs si-NC

(A) 5-Ethynyl-20-deoxyuridine assay (EDU) assays to detect the proliferation of PASMCs in each group in hypoxia (n=3 experiments). (B) Transwell migration experiment to detect the migration of PASMCs in each group in hypoxia (n=3 experiments). (C) Western blot was used to detect the inhibition of Cited2 on the negative regulation of TGF-β1 on peroxisome proliferatoractivated receptor gamma (PPARγ) in PASMCs in hypoxia (n=3 experiments). Data are means±SEM, ^#^ *P<*0.05, ^## ^*P<*0.01, **P<0.01 vs Control

(A) 5-Ethynyl-20-deoxyuridine assay (EDU) assays to detect the proliferation of PASMCs in each group in hypoxia (n=3 experiments). (B) Transwell migration experiment to detect the migration of PASMCs in each group in hypoxia (n=3 experiments). Data are means±SEM, #*P<*0.05, ^##^*P<*0.01, **P<0.01 vs Control

## Discussion

Hypoxic pulmonary vasoconstriction, pulmonary arterial remodeling, and other cases result in HPH (40). Pulmonary vascular remodeling, which is related to pathological changes, such as dysfunction of PASMCs, fibroblast, and pulmonary artery endothelial cells, is a pathological feature of HPH (2). The increased proliferation and migration of PASMCs considerably enhance the process leading to pulmonary artery remodeling (41). 

Current studies have proven the functions of Cited2 in the apoptosis and proliferation of endothelial cells, cardiomyocytes, cancer cells, neuronal cells, and hematopoietic stem cells (9, 12, 42). However, no research has been conducted on the expression of Cited2 in HPH and its effect on PASMC physiological function in hypoxia. In this study, Cited2 was speculated to possibly regulate the migration and proliferation of hypoxia-induced PASMCs. So, the connection between Cited2 and cell migration and proliferation in hypoxia, as well as the possible signaling pathways were investigated. The central findings were as follows: (i) Cited2 expression was inhibited in hypoxia, (ii) Cited2 inhibited the proliferation and migration of PASMCs in hypoxia, (iii) TGF-β1 negatively regulated the expression of Cited2, (iv) Cited2 reversed the TGF-β1-induced proliferation and migration of PASMCs in hypoxia, (v) Cited2 inhibited the negative regulation of the TGF-β1 pathway on PPARγ in hypoxia, and (vi) PPARγ cooperates with Cited2 to inhibit the proliferation and migration of PASMCs. Collectively, these findings showed that Cited2 regulates the proliferation and migration of PASMCs by inhibiting the negative regulation of the TGF-β1 pathway on PPARγ.

Cited2 has been shown to be involved in regulating hypoxia response in certain diseases (43-45). A study also reported that Cited2 levels apparently decreased after 4 hr of hypoxia (46). The same results were obtained in mammalian macrophages and blunt snout bream adult tissues (30, 31). Meanwhile, this study proved that hypoxia down-regulated the Cited2 level in PASMCs and the pulmonary arteries of rats, indicating that Cited2 may be crucial for controlling cellular responses to hypoxia. 

Cited2 serves as a molecular switch of cytokine-induced proliferation and apoptosis in various cells (10, 25, 47). It plays also a meaningful role in lung development (48). In this study, lentivirus was used to overexpress or knock down Cited2 to further explore the relationship between Cited2 and PASMC proliferation and migration. The outcomes showed that PASMCs’ migration and proliferation were significantly inhibited by Cited2 overexpression in hypoxia, but the effect was reversed when Cited2 was knocked down. These findings revealed that Cited2 is a hypoxia-responsive gene, and Cited2-mediated inhibition of PASMC proliferation and migration probably plays a significant role in the suppression of pulmonary arterial remodeling, thereby providing evidence for Cited2 as a novel treatment target for HPH in the future.

Although Cited2 may participate in the development of HPH, its expression was inhibited in hypoxia. Literature was reviewed to further confirm the reasons for Cited2 down-regulation under prolonged hypoxia, and the Endings displayed that TGF-β1 could down-regulate Cited2 expression. TGF-β1 is involved in PH origin and development by inducing the proliferation and migration of PASMCs (49, 50). Interestingly, Cited2 down-regulation was associated with elevated TGF-β1 expression in hypoxias. This result indicated that Cited2 is a TGF-β1-responsive gene in hypoxia and may regulate the TGF-β1 signaling pathway. The effect of TGF-β1 on the formation of HPH may also be related to the inhibition of Cited2 expression.

This study importantly showed that Cited2 reversed the TGF-β1-induced proliferation and migration of PASMCs. However, the precise mechanism by which Cited2 regulates these cellular processes of PASMCs is unclear and needs further exploration. The results displayed that TGF-β1 down-regulated Cited2 expression, and Cited2 inhibited the proliferation and migration of PASMCs in hypoxia, revealing that it may be involved in TGF-β1 signal transduction to regulate cell proliferation and migration. According to previous studies, Cited2 was co-expressed with PPARα and PPARγ in mouse tissues and was required for PPARγ activation (31, 38, 51). PPARγ has been proven to exert the anti-proliferative function of PASMCs (52), and it was down-regulated in HPH animal models and hypoxic PASMCs (36, 52). TGF-β1 could promote PASMC proliferation by inhibiting the activation of the downstream target gene PPARγ (53). Moreover, PPARγ could transrepressed TGF-β1 (54) and inhibit TGF-β1-induced VSMC proliferation (37). Thus, the present study speculated that during hypoxia, Cited2 could inhibit the negative regulation of the TGF-β1 pathway on PPARγ. Following Cited2 overexpression in PASMCs, the inhibitory effect of TGF-β1 on PPARγ was found to be partially eliminated. To further demonstrate that PPARγ cooperates with Cited2 to inhibit the proliferation and migration of PASMCs, we used PPARγ inhibitor in Cited2-overexpressed PASMCs in hypoxia. The results revealed that the ability of Cited2 to inhibit cell proliferation and migration was attenuated by PPARγ deficiency. In summary, Cited2 inhibits the negative regulation of the TGF-β1 pathway on PPARγ and cooperates with PPARγ, consequently affecting the proliferation and migration of PASMCs in hypoxia. 

## Conclusion

This study showed that Cited2 has the function of inhibiting the proliferation and migration of PASMCs in hypoxia through the TGF-β1/Cited2/PPARγ pathway. The results may have a certain guiding importance for targeted therapy of patients with HPH in the future.

## Authors’ Contributions

H W Experiments performance , Data curation, Writing-original draft. L M Validation, Funding acquisition, Writing-original draft. Q Y Supervision, Funding acquisition, Writing-Reviewing and Editing. All of the authors final approved of the version to be published.

## Conflicts of Interest

The authors declare no conflicts of interest. 

## References

[1] Galie N, Humbert M, Vachiery JL, Gibbs S, Lang I, Torbicki A (2015). 2015 ESC/ERS Guidelines for the diagnosis and treatment of pulmonary hypertension: The Joint Task Force for the Diagnosis and Treatment of Pulmonary Hypertension of the European Society of Cardiology (ESC) and the European Respiratory Society (ERS): Endorsed by: Association for European Paediatric and Congenital Cardiology (AEPC), International Society for Heart and Lung Transplantation (ISHLT). Eur Respir J.

[2] Nathan SD, Barbera JA, Gaine SP, Harari S, Martinez FJ, Olschewski H (2019). Pulmonary hypertension in chronic lung disease and hypoxia. Eur Respir J.

[3] Wilkins MR, Ghofrani HA, Weissmann N, Aldashev A, Zhao L (2015). Pathophysiology and treatment of high-altitude pulmonary vascular disease. Circulation.

[4] Penumatsa KC, Singhal AA, Warburton RR, Bear MD, Bhedi CD, Nasirova S (2022). Vascular smooth muscle ROCK1 contributes to hypoxia-induced pulmonary hypertension development in mice. Biochem Biophys Res Commun.

[5] Shi Y, Jiang R, Qin X, Gao A, Hou X, Chen L (2021). Up-regulation of nPKC contributes to proliferation of mice pulmonary artery smooth muscle cells in hypoxia-induced pulmonary hypertension. Eur J Pharmacol.

[6] Imakawa K, Dhakal P, Kubota K, Kusama K, Chakraborty D, Karim Rumi MA (2016). CITED2 modulation of trophoblast cell differentiation: insights from global transcriptome analysis. Reproduction.

[7] Fernandes MT, Calado SM, Mendes-Silva L, Braganca J (2020). CITED2 and the modulation of the hypoxic response in cancer. World J Clin Oncol.

[8] Wu Q, Liu Q, Zhan J, Wang Q, Zhang D, He S (2019). Cited2 regulates proliferation and survival in young and old mouse cardiac stem cells. BMC Mol Cell Biol.

[9] Wu ZZ, Sun NK, Chao CC (2011). Knockdown of CITED2 using short-hairpin RNA sensitizes cancer cells to cisplatin through stabilization of p53 and enhancement of p53-dependent apoptosis. J Cell Physiol.

[10] Gonzalez YR, Zhang Y, Behzadpoor D, Cregan S, Bamforth S, Slack RS (2008). CITED2 signals through peroxisome proliferator-activated receptor-gamma to regulate death of cortical neurons after DNA damage. J Neurosci.

[11] Wang X, Lockhart SM, Rathjen T, Albadawi H, Sorensen D, O’Neill BT (2016). Insulin down-regulates the transcriptional coregulator CITED2, an inhibitor of proangiogenic function in endothelial cells. Diabetes.

[12] Clark AL, Naya FJ (2015). MicroRNAs in the myocyte enhancer factor 2 (MEF2)-regulated Gtl2-Dio3 noncoding RNA locus promote cardiomyocyte proliferation by targeting the transcriptional coactivator cited2. J Biol Chem.

[13] Machado-Oliveira G, Guerreiro E, Matias AC, Facucho-Oliveira J, Pacheco-Leyva I, Braganca J (2015). FBXL5 modulates HIF-1alpha transcriptional activity by degradation of CITED2. Arch Biochem Biophys.

[14] Glenn DJ, Maurer RA (1999). MRG1 binds to the LIM domain of Lhx2 and may function as a coactivator to stimulate glycoprotein hormone alpha-subunit gene expression. J Biol Chem.

[15] Braganca J, Eloranta JJ, Bamforth SD, Ibbitt JC, Hurst HC, Bhattacharya S (2003). Physical and functional interactions among AP-2 transcription factors, p300/CREB-binding protein, and CITED2. J Biol Chem.

[16] Mattes K, Berger G, Geugien M, Vellenga E, Schepers H (2017). CITED2 affects leukemic cell survival by interfering with p53 activation. Cell Death Dis.

[17] Zhang X, Chen W, Liu W, Li D, Shen W (2022). CITED2 alleviates lipopolysaccharide-induced inflammation and pyroptosis in-human lung fibroblast by inhibition of NF-kappaB pathway. Allergol Immunopathol (Madr).

[18] Chu WT, Chu X, Wang J (2020). Investigations of the underlying mechanisms of HIF-1alpha and CITED2 binding to TAZ1. Proc Natl Acad Sci U S A.

[19] Wang RR, Yuan TY, Chen D, Chen YC, Sun SC, Wang SB (2022). Dan-shen-yin granules prevent hypoxia-induced pulmonary hypertension via STAT3/HIF-1alpha/VEGF and FAK/AKT Signaling Pathways. Front Pharmacol.

[20] Chen X, He Y, Yu Z, Zuo J, Huang Y, Ruan Y (2022). Polydatin glycosides improve monocrotaline-induced pulmonary hypertension injury by inhibiting endothelial-to-mesenchymal transition. Front Pharmacol.

[21] Ruiz-Ortiz I, De Sancho D (2020). Competitive binding of HIF-1alpha and CITED2 to the TAZ1 domain of CBP from molecular simulations. Phys Chem Chem Phys.

[22] Sanada TJ, Sun XQ, Happe C, Guignabert C, Tu L, Schalij I (2021). Altered TGFbeta/SMAD signaling in human and rat models of pulmonary hypertension: An old target needs attention. Cells.

[23] Cao N, Liu X, Tang X, Gao R, Ma K, Li L (2022). [Emodin inhibits the proliferation and migration of human pulmonary artery smooth muscle cells by blocking SMAD2/3 signaling pathway]. Xi Bao Yu Fen Zi Mian Yi Xue Za Zhi..

[24] Hu C, Zhang Y, Tang K, Luo Y, Liu Y, Chen W (2017). Down-regulation of CITED2 contributes to TGFbeta-mediated senescence of tendon-derived stem cells. Cell Tissue Res.

[25] Chou YT, Hsieh CH, Chiou SH, Hsu CF, Kao YR, Lee CC (2012). CITED2 functions as a molecular switch of cytokine-induced proliferation and quiescence. Cell Death Differ.

[26] Chou YT, Yang YC (2006). Post-transcriptional control of Cited2 by transforming growth factor beta Regulation via Smads and Cited2 coding region. J Biol Chem.

[27] Jayaraman S, Doucet M, Kominsky SL (2017). Down-regulation of CITED2 attenuates breast tumor growth, vessel formation and TGF-beta-induced expression of VEGFA. Oncotarget.

[28] Chou YT, Wang H, Chen Y, Danielpour D, Yang YC (2006). Cited2 modulates TGF-beta-mediated upregulation of MMP9. Oncogene.

[29] Liu HL, Yu D, Zhu ZN, Su SW, Chen XY, Zhang YJ (2015). m-Nisodipine inhibited 5-HT-induced proliferation of rat PASMCs through Rho/ROCK signal pathway. Yao Xue Xue Bao.

[30] Sun Y, Guo HH, Guo DD, Jiang XY, Zou SM (2018). Divergence of genes encoding CITED1 and CITED2 in blunt snout bream (megalobrama amblycephala) and their transcriptional responses to hypoxia. Front Physiol.

[31] Kim GD, Das R, Rao X, Zhong J, Deiuliis JA, Ramirez-Bergeron DL (2018). CITED2 restrains proinflammatory macrophage activation and response. Mol Cell Biol.

[32] Luo X, Ding L, Xu J, Chegini N (2005). Gene expression profiling of leiomyoma and myometrial smooth muscle cells in response to transforming growth factor-beta. Endocrinology.

[33] Kang Y, Chen CR, Massague J (2003). A self-enabling TGFbeta response coupled to stress signaling: Smad engages stress response factor ATF3 for Id1 repression in epithelial cells. Mol Cell.

[34] Zhang Y, Yuan RX, Bao D (2020). TGF-beta1 promotes pulmonary arterial hypertension in rats via activating RhoA/ROCK signaling pathway. Eur Rev Med Pharmacol Sci.

[35] Cai G, Liu J, Wang M, Su L, Cai M, Huang K (2019). Mutual promotion of FGF21 and PPARgamma attenuates hypoxia-induced pulmonary hypertension. Exp Biol Med.

[36] Gong K, Xing D, Li P, Aksut B, Ambalavanan N, Yang Q (2011). Hypoxia induces down-regulation of PPAR-gamma in isolated pulmonary arterial smooth muscle cells and in rat lung via transforming growth factor-beta signaling. Am J Physiol Lung Cell Mol Physiol.

[37] Calvier L, Chouvarine P, Legchenko E, Hoffmann N, Geldner J, Borchert P (2017). PPARgamma links BMP2 and TGFbeta1 pathways in vascular smooth muscle cells, regulating cell proliferation and glucose metabolism. Cell Metab.

[38] Tien ES, Davis JW, Vanden Heuvel JP (2004). Identification of the CREB-binding protein/p300-interacting protein CITED2 as a peroxisome proliferator-activated receptor alpha coregulator. J Biol Chem.

[39] Victor Tseng, Roy L Sutliff, C Michael Hart (2019). Redox biology of peroxisome proliferator-activated receptor-γ in pulmonary hypertension. Antioxid Redox Signal.

[40] Kylhammar D, Radegran G (2017). The principal pathways involved in the in vivo modulation of hypoxic pulmonary vasoconstriction, pulmonary arterial remodelling and pulmonary hypertension. Acta Physiol (Oxf).

[41] Christou H, Khalil RA (2022). Mechanisms of pulmonary vascular dysfunction in pulmonary hypertension and implications for novel therapies. Am J Physiol Heart Circ Physiol.

[42] Liu YC, Chang PY, Chao CC (2015). CITED2 silencing sensitizes cancer cells to cisplatin by inhibiting p53 trans-activation and chromatin relaxation on the ERCC1 DNA repair gene. Nucleic Acids Res.

[43] Niwa T (2011). Role of indoxyl sulfate in the progression of chronic kidney disease and cardiovascular disease: experimental and clinical effects of oral sorbent AST-120. Ther Apher Dial.

[44] Yang SL, Liu LP, Niu L, Sun YF, Yang XR, Fan J (2016). Down-regulation and pro-apoptotic effect of hypoxia-inducible factor 2 alpha in hepatocellular carcinoma. Oncotarget.

[45] Tanaka T, Yamaguchi J, Higashijima Y, Nangaku M (2013). Indoxyl sulfate signals for rapid mRNA stabilization of Cbp/p300-interacting transactivator with Glu/Asp-rich carboxy-terminal domain 2 (CITED2) and suppresses the expression of hypoxia-inducible genes in experimental CKD and uremia. FASEB J.

[46] Beucken T, Magagnin MG, Savelkouls K, Lambin P, Koritzinsky M, Wouters BG (2007). Regulation of Cited2 expression provides a functional link between translational and transcriptional responses during hypoxia. Radiother Oncol.

[47] Lou X, Sun S, Chen W, Zhou Y, Huang Y, Liu X (2011). Negative feedback regulation of NF-kappaB action by CITED2 in the nucleus. J Immunol.

[48] Xu B, Qu X, Gu S, Doughman YQ, Watanabe M, Dunwoodie SL (2008). Cited2 is required for fetal lung maturation. Dev Biol.

[49] Cao N, Aikeremu N, Shi WY, Tang XC, Gao RJ, Kong LJ (2022). Inhibition of KIR2 1 decreases pulmonary artery smooth muscle cell proliferation and migration. Int J Mol Med.

[50] Cao N, Tang X, Gao R, Kong L, Zhang J, Qin W (2021). Galectin-3 participates in PASMC migration and proliferation by interacting with TGF-beta1. Life Sci.

[51] Liu Z, Wang Y, Dou C, Sun L, Li Q, Wang L (2018). MicroRNA-1468 promotes tumor progression by activating PPAR-gamma-mediated AKT signaling in human hepatocellular carcinoma. J Exp Clin Cancer Res.

[52] Zhang Q, Feng W, Wang Q, Wang J, Chai L, Chen Y (2022). PPARγ activation inhibits PDGF-induced pulmonary artery smooth muscle cell proliferation and migration by modulating TERT. Biomed Pharmacother.

[53] Liu Y, Cao Y, Sun S, Zhu J, Gao S, Pang J (2016). Transforming growth factor-beta1 upregulation triggers pulmonary artery smooth muscle cell proliferation and apoptosis imbalance in rats with hypoxic pulmonary hypertension via the PTEN/AKT pathways. Int J Biochem Cell Biol.

[54] Pan K, Lu J, Song Y (2021). Artesunate ameliorates cigarette smoke-induced airway remodelling via PPAR-gamma/TGF-beta1/Smad2/3 signalling pathway. Respir Res.

